# Supplementary material for: Topical Delivery of Ketorolac Tromethamine via Cataplasm for Inflammatory Pain Therapy
Source: Pharmaceutics. 2023 May 4;15(5):1405. doi: 10.3390/pharmaceutics15051405 (PMC10223623; doi:10.3390/pharmaceutics15051405)
Supplement: Supplementary file 1 [file pharmaceutics-15-01405-s001.zip › pharmaceutics-2293675-supplementary.pdf]

Article

Topical Delivery of Ketorolac Tromethamine via Cataplasm for Inflammatory Pain Therapy

Zhiyuan Hou [1,†], Qiang Wen [1,†], Wenhu Zhou [1], Peng Yan [1], Hailong Zhang [1,2,*] and Jinsong Ding [1,*]

[1] Xiangya School of Pharmaceutical Science, Central South University, Changsha, Hunan 410006, China
[2] Changsha Jingyi Pharmaceutical Technology Co., LTD, Changsha, Hunan 410006, China
* Correspondence: zhl@king-eagle.cn (H.Z.); jinsongding@hotmaill.com (J.D.)
† These authors contributed equally to this work.

Supplementary Data:

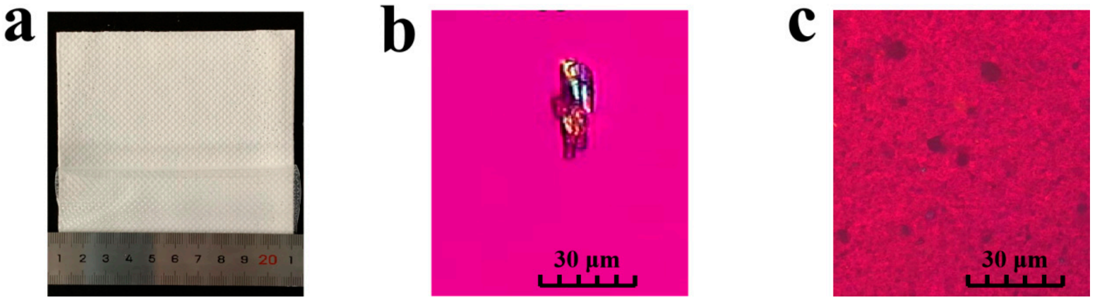

Figure S1. Appearance and polarization microscopic characterization of cataplasm. **(a)** Appearance of the cataplasm; **(b)** Polarized light microscopic observation of ketorolac tromethamine (200X); **(c)** Polarized light microscopic observation of the cataplasm paste (200X).

Table S1. Cumulative drug release amount of different doses of cataplasm (n = 6).

**Dose**	**Cumulative drug release amount (%)**									
**(%)**	**0.25 h**	**0.5 h**	**0.75 h**	**1 h**	**2 h**	**4 h**	**6 h**	**8 h**	**10 h**	**12 h**
0.5	15.03	21.51	27.10	31.90	48.66	70.26	87.00	92.00	95.83	97.38
1.0	13.89	20.49	25.85	31.79	46.85	67.54	83.76	90.41	93.99	97.86
1.5	15.80	22.56	28.19	33.04	50.16	69.20	85.31	91.36	96.11	96.67

Table S2. Summary of demographic characteristics of the subjects (n = 8).

**Age (year)**	**Height (cm)**	**Weight (kg)**	**BMI (kg/m^2)**
24.57 ± 2.94	168.60 ± 5.15	62.50 ± 7.54	22.00 ± 2.58

All data are expressed as mean ± SD.
